# The generation that lived during/participated in the war and the generation that inherited it: association between veterans PTSD and adult offspring’s emotional regulation strategies and alexithymia levels

**DOI:** 10.1186/s12888-023-05087-y

**Published:** 2023-08-17

**Authors:** Perla El Moujabber, Vanessa Homsi, Souheil Hallit, Sahar Obeid

**Affiliations:** 1 School of Arts and Sciences, Psychology Department, Holy Spirit University of Kaslik, P.O. Box 446, Jounieh, Lebanon; 2 School of Arts and Sciences, Social and Education Sciences Department, Lebanese American University, Jbeil, Lebanon; 3 School of Medicine and Medical Sciences, Holy Spirit University of Kaslik, P.O. Box 446, Jounieh, Lebanon; 4 Applied Science Research Center, Applied Science Private University, Amman, Jordan; 5 Research Department, Psychiatric Hospital of the Cross, Jal Eddib, Lebanon

**Keywords:** Post-traumatic stress disorder, Lebanese civil war, Veterans, Offspring, Emotional regulation, Alexithymia

## Abstract

**Background:**

The long-term repercussions that war can have on both war generations and post-war generations lack in the literature. It is imperative to understand the psychological consequences of the Lebanese Civil War that took place from 1975 to 1990, on the offspring of those who took part in it. Accordingly, the objective of this study was to assess the association between paternal/veterans PTSD and adult offspring’s emotional regulation strategies and alexithymia levels, 30 years after the end of war.

**Method:**

A cross-sectional study was carried out between September 2020 and September 2021 on a sample of 75 fathers of Lebanese former veterans and paramilitary veterans and their adult offspring. For the veterans and paramilitary veterans’ population, the PTSD Checklist was used to assess post-traumatic stress disorder, and the Combat Exposure Scale (CES) was used to measure the level of combat exposure. For the offspring population, the Emotional Regulation Questionnaire (ERQ) was used to assess emotional regulation strategies and the Toronto Alexithymia Scale (TAS) was used to measure the levels of alexithymia.

**Results:**

Paternal PTSD (Beta = 10.19) was associated with higher levels of alexithymia in the offspring population. Regarding emotional regulation strategies, results showed that paternal PTSD (Beta = -3.24) was significantly associated with a decrease in the cognitive reappraisal score in the offspring. Also, paternal PTSD (Beta = 4.57) was significantly associated with an increase in expressive suppression score. Additionally, an older father’s age (Beta = 1.11) was significantly associated with an increased alexithymia score in the offspring. Moreover, results showed that paternal combat injuries (Beta = -4.24) were significantly associated with a decrease in the alexithymia score in the offspring population and an increase in the expressive suppression score (Beta = 3.28).

**Conclusion:**

This study shows that fathers’ traumatic experience of war influences emotion regulation and alexithymia levels in their offspring. Longitudinal studies taking into account the age of the offspring at the time of onset of fathers’ symptoms may provide us with additional information to understand the influence of paternal PTSD on the emotional functioning of offspring during different phases of emotional development.

## Introduction

Post-Traumatic Stress Disorder (PTSD) is a psychiatric disorder that can occur in people across all ages, cultures, or gender who have experiences or witnessed a traumatic event [1]. In a military context, trauma can occur following a threat to life through participation in armed combat or other military activities: patrols, espionage, and dangerous tasks. War-related traumatic events constitute the highest conditional risk for the development of PTSD [2]. The risk of developing PTSD in the military context depends on several factors, and largely depends on the level of stress and social support received after deployment [3].

Deployment to war primarily occurs in early adulthood, which is normally the time when combatants start their families, making the offspring population particularly vulnerable to the effects of war-related trauma. This vulnerability could be a consequence of altered combatant behavior, psychopathology regarding parenting, or secondary trauma [4]. In this line, PTSD can lead to negative alterations in an individual’s behavior, including increased anger and reactivity, as well as social withdrawal [1]. For example, the tendency to rely on strategies such as emotional numbing appears to have a ‘particularly detrimental effect on the quality of the veteran-child relationship’ [5]. Growing up in an aggressive, stressful and unpredictable family environment can have detrimental consequences on the future life of the child [6].

Characteristics of emotional numbness, such as unavailability, detachment, and disinterest as well as difficulty experiencing positive emotions, are closely linked to interpersonal impairment which consequently reduces a parent’s ability to participate and enjoy interactions with their child, thus diminishing the possibility of establishing a meaningful relationship [7]. Furthermore, withdrawal, isolation, inability to express emotions [5, 7], overprotection and excessive control of the child [8] are some of the difficulties experienced by veterans when trying to function within the family system. In such family environments, important child developmental processes are particularly disrupted, such as attachment, separation, and individualization [9]. Childhood and adolescence are particularly critical developmental stages [10], and disruptions experienced at these stages affect subsequent education, socialization and functioning in adulthood.

As extensive research points out, the trauma of fighters (or parental PTSD) can impact their offspring, which can lead to later developmental, psychological, emotional, behavioral, and social difficulties [11–15]. One of these long-term impacts that need to be studied are dysfunctional emotions such as emotional identification and regulation.

On one hand, Gross and colleagues [16, 17] focused on two emotional regulation strategies: cognitive reappraisal (antecedent-focused strategy) and expressive suppression (response-focused strategy). Reappraisal is an antecedent-focused strategy that aims to alter the emotional meaning and impact of an emotionally eliciting situation [18]. In contrast, suppression is a form of response modulation defined as inhibiting emotional expression [19]. As suppression occurs later in the emotion generation process, it does not influence the emotion itself, but rather its outcomes [20]. Emotion regulation supports psychological health and well-being, and helps manage negative life events and stress [21–23]. Morris et al. [24] proposed that parents provide role models through which their children mimic their emotional regulation strategies. Parents with psychopathology, including dysregulated emotions, may not be adequate models for their children to acquire emotion regulation [25]. Despite their less frequent involvement compared to mothers, fathers also play a central and irreplaceable role in socializing children’s emotional regulation abilities [26]. Thus, the offspring of veterans with PTSD might have a high probability of manifesting emotional deficits.

The development of emotion regulation is also influenced by the emotional climate of the family, attachment and marital relationships [24]. In addition to having emotional regulation dysfunction, veteran parents with PTSD also have family dysfunction issues in areas such as conflict, family cohesion, marital adjustment, offspring abuse, affective reactivity and problem solving, where the latter two directly affect the development of children’s emotion regulation [27–29].

On the other hand, Sifneos (1972) [30] defines alexithymia as a deficit of affect which results in a difficulty in identifying, understanding, and communicating the emotions of oneself and those of others. Today, its definition is more explicitly refined with five dominant features: (1) difficulty in identifying one’s emotions and being able to distinguish them from one’s bodily sensations (somatic complaints are very frequent ); (2) verbal difficulty in describing feelings to others; (3) a reduction or inability to feel emotions; (4) a lack of tendency to imagine another’s emotion, or a cognitive style oriented outwardly rather than inwardly; and (5) low capacity for fantasy or symbolic thought [31]. This emotional regulation disorder, whose origin, role, and function may vary, could result from an imitation of the parents’ way of managing their emotions, or conversely, the adoption of a different attitude to that used by parents, such as compensating in adulthood for the lack of emotional sharing experienced in childhood [32].

People with alexithymia have difficulty regulating their emotions. Low level of emotion regulation is associated with low levels of social ability, emotional expression, and emotional intelligence [33]. Several models have been proposed regarding the etiology of alexithymia, some theorists hypothesize that childhood events such as traumatic experiences (mourning, separation, or other events) and/or a dysfunctional parent-child relationship contribute to alexithymia [32]. Some research has also indicated that optimal parenting in one parent may protect against the development of alexithymia even if parenting in the other parent is perceived to be more pathological [34].

From 1975 to 1990, a multidimensional civil war affected Lebanon [35]. In 1975, began a period of struggles and massacres between armed forces from several political and religious components. These deadly conflicts set the country on fire and bloodshed for fifteen years [35]. An unknown number of civilian Lebanese men turned fighters took part in the fighting. It is imperative to understand the psychological consequences of war on the offspring, even if many health problems of veterans remain to be studied. Accordingly, the objective of this study was to assess the association between paternal/veterans PTSD and adult offspring’s emotional regulation strategies and alexithymia levels, assessed 30 years after the end of war. We hypothesized that combatants’ PTSD would be associated with emotional dysregulation (lower cognitive reappraisal, higher expressive suppression, and alexithymia), when assessed 30 years after their fathers’ exposure to war-related trauma.

## Methods

### Study design

A cross-sectional study was carried out between September 2020 and September 2021, and enrolled 150 participants (a sample of 75 fathers of Lebanese former veterans and paramilitary veterans and their adult offspring), recruited from the general population chosen in a convenient way from all Lebanese governorates (Beirut, Mount Lebanon, North Lebanon, South Lebanon, and Bekaa). Lebanese veterans were selected from the general population, a non-clinical sample of men who participated in or lived during the Lebanese war 30 years ago. Invitations to participate in the study were sent through social media platforms, acquaintances and specifically political parties to find veterans who participated in the war; veterans are more likely to be affiliated to political parties than are those of comparable ages who are not veterans [36]. After that, we used the snowball sampling technique (each subject provided multiple referrals) to recruit the rest of the veteran-offspring sample.

Data was collected from each participant through face-to-face personal interviews after obtaining their oral consent. These interviews were conducted by a clinical psychologist, who received a thorough training by the principal investigator on how to ask the questions and how to communicate with the patients.

### Minimal sample size calculation

We used the G*Power software to determine the sample size. The minimum required sample size was 70 participants, considering an alpha error of 5%, a power of 80%, a minimal model R-square of 0.25 and allowing 20 predictors to be included in the model.

### Questionnaire

The questionnaire used was anonymous and in Arabic, the native language in Lebanon; it required approximately 15 min to complete. The questionnaire consisted of three parts. The first part of the questionnaire included an explanation of the study topic and objective, and a statement ensuring the anonymity of respondents.

The second part of the questionnaire contained sociodemographic information about the father (age, educational level, marital status), and adult offspring (age, sex, marital status and educational level). We also collected data related to combat injuries (casualties to military personnel resulting from combat) and physical injuries (chronic illnesses that have resulted from being exposed to war and/or prolonged combat) among veterans. For the veterans and paramilitary veterans’ population, the PTSD Checklist was used to assess post-traumatic stress disorder, and the Combat Exposure Scale (CES) was used to measure the level of combat exposure. For the offspring population, the Emotional Regulation Questionnaire (ERQ) was used to assess emotional regulation strategies and the Toronto Alexithymia Scale (TAS) was used to measure the levels of alexithymia.

The third part included the scales used in this study:

**PTSD Checklist.** This questionnaire was used to evaluate the manifestations of PTSD according to the DSM-4 [37]. It has 17 items that are scored from 1 (not at all) to 5 (extremely); higher results imply greater severity of PTSD symptoms [38] (Cronbach’s alpha in this study = 0.92).

**Combat Exposure Scale** (CES) is a 7-item self-report measure that assesses wartime stressors experienced by combatants. Items are rated on a 5-point frequency (1 = “no” or “never” to 5 = “26 + times” or “51 + times”), 5-point duration (1 = “never” to 5 = “7 + months”), or 45-point degree of loss (1 = “none” to 45 = “76% or more”) scale. The total CES score (ranging from 0 to 41) is calculated by using a sum of weighted scores, which can be classified into one of five categories of combat exposure ranging from “light” to “heavy” [39] (Cronbach’s alpha in this study = 0.92).

**Emotion Regulation Questionnaire.** Validated in Lebanon [40], it is composed of 10 items that measure whether a respondent uses cognitive reappraisal or expressive suppression to regulate their emotions. Answers options varied between 1 (strongly disagree) and 7 (strongly agree). Higher scores reflect a larger use of the concerned emotion regulation strategy [18] (Cronbach’s alpha in this study = 0.85 for expressive suppression and 0.74 for cognitive reappraisal).

**Toronto Alexithymia Scale (TAS-20).** Validated in Lebanon [41], this 20-item scale was used to assess alexithymia [42]. Items are rated using the 5-point Likert scale from 1 = strongly disagree to 5 = strongly agree. Participants scoring ≤ 51 were classified as non-alexithymic, whereas those scoring between 52, 60 and ≥ 61 were classified as being possibly alexithymic and alexithymic respectively (Cronbach’s alpha in this study = 0.77).

### Translation procedure

The forward and backward translation method was applied to different scales (PTSD Checklist and Combat Exposure Scale). The English version was translated to Arabic by a Lebanese translator who was completely unrelated to the study. Afterwards, a Lebanese psychologist with a full working proficiency in English, translated the Arabic version back to English. The initial and translated English versions were compared to detect and later eliminate any inconsistencies.

### Statistical analysis

SPSS version 25 software was used for statistical analysis. The sample was normally distributed as verified by the skewness and kurtosis values of the alexithymia, cognitive reappraisal and expressive suppression [43]. Linear regressions were conducted, taking alexithymia, cognitive reappraisal and expressive suppression scores as dependent variables. The results of the multivariate analyzes were adjusted for the following independent variables: father’s exposure to combat, father’s PTSD, father’s age, father’s education, physical injuries during the war, father suffered combat injuries, as well as age, sex and level of education of the offspring. Significance was set at p < .05.

## Results

A total of 75 fathers and 75 adult offspring participated in this study. The average age of the fathers was 65.37 ± 6.20 years, while that of the adult offspring was 38.49 ± 7.45 years. The majority of the adult offspring were men (66.7%). Other participant characteristics can be found in Table 1.

**Table 1:** Sociodemographic characteristics of the participants

Variable	Parents (N = 75)	Adult Offspring (N = 75)
**Sex**		
Men	75 (100.0%)	50 (66.7%)
Women	0 (0%)	25 (33.3%)
**Marital Status**		
Married	74 (98.7%)	56 (74.7%)
Single / Divorced	1 (1.3%)	19 (25.3%)
**Education level**		
Primary or less	14 (18.7%)	-
Complementary	10 (13.3%)	-
Secondary	34 (45.3%)	11 (14.7%)
University	17 (22.7%)	64 (85.3%)
**Physical injuries during the war (yes)**	6 (8.0%)	-
**Combat injuries (yes)**	60 (80.0%)	-
	**Mean ± SD**	**Mean ± SD**
**Age (in years)**	65.37 ± 6.20	38.49 ± 7.45
**Father’s exposure to combat**	27.08 ± 3.11	-
**Father’s PTSD**	32.53 ± 7.58	-
**Alexithymia score**	-	52.16 ± 8.03
**Cognitive reappraisal score**	-	26.36 ± 3.73
**Expressive suppression score**	-	14.31 ± 3.96

The results showed that 30.7% of participants (adult offspring) had probable alexithymia while 18.70% of participants had alexithymia. On the other hand, 6.7% of the participants had mild PTSD, 61.3% moderate PTSD and 1.3% severe PTSD.

### Bivariate analysis

The results of the bivariate analysis are summarized in Tables 2 and 3.

**Table 2:** Bivariate analysis of categorical variables associated with alexithymia, cognitive reappraisal and expressive suppression

	Alexithymia	Cognitive reappraisal	Expressive suppression
**Education level of the father**			
Primary or less	43.71 ± 6.81	26.90 ± 3.37	13.19 ± 4.02
Complementary	44.95 ± 7.07	26.45 ± 3.13	13.14 ± 3.20
Secondary	44.53 ± 7.39	28.09 ± 3.77	12.74 ± 3.82
University	44.46 ± 6.88	27.65 ± 4.15	13.31 ± 4.17
p	0.952	0.320	0.929
**Being a warrior during the war**			
No	40.24 ± 2.63	29.49 ± 2.62	10.68 ± 1.92
Yes	46.75 ± 7.62	26.36 ± 3.73	14.31 ± 3.96
*p*	**< 0.001**	**< 0.001**	**< 0.001**
**Physical illnesses during and after the war**			
No	44.21 ± 6.89	27.51 ± 3.69	12.91 ± 3.65
Yes	46.73 ± 8.30	27.00 ± 3.85	14.09 ± 5.01
*p*	0.261	0.662	0.330
**Suffered combat injuries in the father**			
No	42.52 ± 6.04	28.46 ± 3.28	11.84 ± 3.32
Yes	46.25 ± 7.46	26.53 ± 3.83	14.13 ± 3.89
*p*	**0.004**	**0.004**	**0.001**
**PTSD in the father**			
No	40.28 ± 2.74	29.25 ± 2.60	10.70 ± 1.97
Yes	49.58 ± 7.33	25.27 ± 3.66	15.88 ± 3.54
*p*	**< 0.001**	**< 0.001**	**< 0.001**
**Sex of the child**			
Men	45.87 ± 7.41	27.14 ± 3.68	13.87 ± 4.14
Women	42.36 ± 5.94	27.94 ± 3.69	11.79 ± 2.81
*p*	**0.006**	0.259	**0.002**
**Marital Status of the child**			
Single	43.44 ± 6.52	27.95 ± 3.72	12.15 ± 3.66
Married	44.96 ± 7.27	27.22 ± 3.67	13.47 ± 3.80
*p*	0.272	0.317	0.078
**Education level of the child**			
Secondary or less	46.82 ± 8.15	26.55 ± 3.24	14.09 ± 4.41
University	44.20 ± 6.91	27.56 ± 3.73	12.91 ± 3.73
*p*	0.242	0.387	0.330

**Table 3:** Correlation matrix of continuous variables

	1	2	3	4	5	6	7
1. Alexithymia	1						
2. Cognitive reappraisal	− 0.61***	1					
3. Expressive suppression	0.80***	− 0.58***	1				
4. Age of the father	0.13	− 0.06	0.11	1			
5. Age of the child	0.14	− 0.11	0.13	0.95***	1		
6. Combat exposure	0.29*	− 0.26*	0.38**	0.21	0.20	1	
7. PTSD score	0.78***	− 0.67***	0.77***	0.14	0.17	0.49***	1

*p < .05; **p < .01; ***p < .001

### Multivariate analysis

The results of a first linear regression, taking the cognitive reappraisal score as the dependent variable, showed that the presence of PTSD in the father (Beta=-3.27) was significantly associated with lower cognitive reappraisal in the offspring (Table 4, Model 1).

The results of a second linear regression, taking the expressive suppression score as the dependent variable, showed that the presence of PTSD in the father (Beta = 4.72) was significantly associated with more expressive suppression in the offspring. Older age of the offspring (Beta = − 0.15) and the gender of the offspring (females vs. males) (Beta= -1.76) were significantly associated with less expressive suppression in the offspring (Table 4, Model 2).

The results of a third linear regression, taking the alexithymia score as the dependent variable, showed that the presence of PTSD in the father (Beta = 0.34) and higher expressive suppression (Beta = 1.04) were significantly associated with higher alexithymia in the offspring (Table 4, Model 3).

**Table 4:** Multivariate analysis

	Beta	β	*P*	95% IC
**Model 1: Cognitive reappraisal score taken as dependent variable (R** ^2^ ** = 0.212)**				
	**Beta**	**β**	***P***	**95% IC**
Father suffered battle injuries (yes vs. no*)	1.11	0.12	0.268	− 0.87; 3.10
Father’s PTSD (yes vs. no*)	-3.27	− 0.41	**0.001**	-5.22; − 0.1.32
Combat exposure scale score	− 0.10	− 0.08	0.519	− 0.39; 0.20
**Model 2: Expressive suppression score taken as dependent variable (R**^**2**^ **= 0.475)**				
Father suffered battle injuries (yes vs. no*)	− 0.89	− 0.09	0.354	-2.80; 1.01
Father’s PTSD (yes vs. no*)	4.72	0.55	**< 0.001**	2.95; 6.49
Combat exposure scale score	0.10	0.08	0.504	− 0.19; 0.38
Offspring sex (female vs. male*)	-1.76	− 0.21	**0.025**	-3.28; − 0.23
Marital status of the offspring (married vs. single*)	1.96	0.22	0.097	− 0.36; 4.28
Age of the offspring	− 0.15	− 0.29	**0.017**	− 0.28; − 0.03
**Model 3: Alexithymia score taken as dependent variable (R**^**2**^ **= 0.699)**				
Father suffered battle injuries (yes vs. no*)	− 0.56	− 0.03	0.691	-3.37; 2.24
Father’s PTSD (yes vs. no*)	0.34	0.32	**0.010**	0.08; 0.59
Offspring sex (female vs. male*)	0.18	0.01	0.888	-2.35; 2.71
Educational level of the offspring (university vs. secondary or less*)	-1.31	− 0.06	0.393	-4.34; 1.73
Cognitive reappraisal	− 0.30	− 0.14	0.143	− 0.70; 0.10
Expressive suppression	1.04	0.51	**< 0.001**	0.59; 1.48
Combat exposure scale score	− 0.27	− 0.10	0.205	− 0.68; 0.15

Numbers in bold indicate significant p values

## Discussion

### Expressive suppression

The results showed that the presence of PTSD in the father was significantly associated with an increase in the expressive suppression score in the offspring. Studies show that people who use expressive suppression more often, generally express fewer positive emotions, have low self-esteem, lower life satisfaction, and greater depressive symptomatology than those who use cognitive reappraisal [18]. Several studies have demonstrated the significant association between parents’ ability to regulate emotions and their children’s emotional regulation skills. Specifically, Bariola, Hughes, and Gullone [44] found that the maternal suppression strategy facilitated children’s use of the suppression strategy. Similarly, Crespo, Trentacosta, Aikins, and Wargo-Aikins [45] found that difficulties in maternal emotion regulation positively predicted children’s emotional regulation deficits. Despite these advances, few studies have investigated the unique contributions of fathers’ emotion regulation skills to their children’s emotion regulation. A growing body of evidence suggests that fathers take on a central and unique role in activating children’s emotional development [46, 47]. Thus, investigating whether the dysregulation of paternal emotions contributed in unique ways to the regulation of children’s emotions beyond the dysregulation of maternal emotions would improve our understanding of the role of fathers as an agent of socialization of emotions.

However, the results showed that the age of the offspring also plays a role in the development of expressive suppression; older age of offspring was significantly associated with less expressive suppression in the offspring. A study conducted on university students in Norway, Australia, and the United States found that the overall mean of people over 25 that use expressive suppression is lower than the mean of people under 25 that use expressive suppression [48]. In addition, the results showed that female offspring was associated with less expressive suppression in the offspring. A study conducted on the correlation between gender and expressive suppression among the Chinese population showed that men score higher than women in expressive suppression [49]. Wang et al. conducted MRI scans in the study and concluded the gender differences were due to differences in cortical thickness in the superior frontal gyrus (SFG) [49]. Interestingly, the same study on university students in Norway, Australia, and the United States also found that as men get older (over 25) they continue to use expressive suppression more than women and scored an overall higher mean on expressive suppression [48].

### Cognitive reappraisal

Concerning emotional regulation strategies, the results showed that the presence of PTSD in the father was significantly associated with a decrease in the cognitive reappraisal score. Previous studies in both Eastern and Western cultures have suggested that cognitive reappraisal reduces negative emotions and increases positive emotions, thereby improving mental health and interpersonal functioning [50, 51]. Many studies indicate that people with PTSD often have problems establishing and maintaining interpersonal relationships in general [52]. A study by Cheung et al. [53] show that greater mother-child and father-child intimacy predicted better cognitive reappraisal and better psychological, social, and general health in a sample of adult offspring. Another study by Wang et al. [54] shows that adolescents whose fathers were supportive and balanced had higher cognitive reappraisal scores than those whose fathers were disengaged and tough. Overall ability to regulate emotions was highest in adolescents with supportive fathers and lowest in adolescents with harsh fathers. Knowing that an individual’s emotional regulation strongly depends on the emotional regulation capacities of his parents, and that this capacity is considered to be transmitted from parents to children [24, 29], we can say that the clinical manifestation of PTSD of the father and the resulting symptoms can influence the regulatory capacities of the offspring favoring less the use of the cognitive reappraisal strategy, which could explain our results.

### Alexithymia

Regarding the alexithymia variable, results showed that the presence of PTSD in the father was associated with more alexithymia in the offspring. Our results are consistent with a previous study by Castro-Vale et al. who showed that PTSD has negative intergenerational effects with respect to emotion regulation, more specifically, that the offspring of veterans with lifelong PTSD 40 years after exposure have impaired recognition of all emotions [15]. More specifically, lifetime PTSD in war veterans 40 years after exposure was associated with impaired ability to identify facial expression of emotions, independent of the type of emotion. Furthermore, the offspring of veterans with lifetime PTSD also showed a reduced ability to identify facial emotional expression both in general, and specifically for the emotions of disgust and happiness when compared with the offspring of veterans without lifetime PTSD [15].

Moreover, results showed that higher expressive suppression was significantly associated with higher alexithymia in the offspring. Previous studies showed that individuals with high alexithymia levels tended to have less effective emotion regulation strategies [55–57]. Additionally, people with high levels of alexithymia tend to use more avoidant and unhelpful emotion regulation strategies, including high expressive suppression. Since emotion dysregulation is a common feature of psychopathologies, it implies that alexithymia may function as a risk factor for psychopathology symptoms primarily through poor emotion regulation [58].

### Clinical implications

Studies regarding the long-term association of veterans’ exposure on adult offspring are lacking in the literature. Lebanon would be a fertile ground to study the repercussions that exposure to war can have on post-war generations. Following our results, veterans and their families should benefit from psycho-educational programs or psychological support, which take into account the long-term psycho-social consequences and the difficulties on a case-by-case basis, and this for the well-being of the individual and the family system.

### Limitations

As with any study, there are several limitations that need to be considered. First, our research follows a cross-sectional plan from which we cannot capture variability over time. For this, longitudinal studies are needed to assess this causal relationship in a more adequate way. Second, there are no studies that compare emotional regulation strategies and alexithymia levels of offspring of warfighters; in fact, research assesses other variables. Third, even if participants are assured of anonymity and confidentiality, they may be tempted to represent themselves positively in such a way that they feel more protected and less exposed. Indeed, the stigma of mental illness in general can lead to inaccurate reporting of symptoms due to fear of privacy breaches. Furthermore, the PTSD Checklist and Combat Exposure Scale used in this study are not yet validated in Lebanon. Additionally, mental disorders of offspring participants, which could affect emotion regulation strategies, was not collected in this current study and could be recommended to be explored in future studies. Finally, our results should be interpreted with caution, due to the possibility of recall bias.

## Conclusion

The statistical results of this study demonstrated that there is a significant positive difference between the levels of alexithymia and the emotional regulation strategies of offspring whose father presents with PTSD and those whose father does not present with PTSD.

In future studies, mothers should also be included, as they have an important moderating role in the relationship between father and offspring, and their mental health status exerts a major influence on offspring psychopathology. Moreover, there is a need to study the longitudinal evolution of fathers and their offspring at the psychopathological level in order to clarify the dynamics of mediation effects over time. Also, the age of the offspring at the onset of the father’s symptoms may provide us with additional information to understand the influence of paternal PTSD on the offspring during different phases of affective development.

## Data Availability

All data generated or analyzed during this study are not publicly available due the restrictions by the ethics committee (data are owned by the Psychiatric Hospital of the Cross). The dataset supporting the conclusions is available upon request to Ms. Rana Nader (rnader@naderlawoffice.com), a member of the ethics committee at the Psychiatric Hospital of the Cross.
